# Cavitating Flow through a Micro-Orifice

**DOI:** 10.3390/mi10030191

**Published:** 2019-03-15

**Authors:** Zhi-jiang Jin, Zhi-xin Gao, Xiao-juan Li, Jin-yuan Qian

**Affiliations:** 1 Institute of Process Equipment, College of Energy Engineering, Zhejiang University, Hangzhou 310027, China; jzj@zju.edu.cn (Z.-j.J.); zhixingao@zju.edu.cn (Z.-x.G.); lixiaojuan@zju.edu.cn (X.-j.L.); 2 Department of Energy Sciences, Lund University, SE-22100 Lund, Sweden; 3 State Key Laboratory of Fluid Power and Mechatronic Systems, Zhejiang University, Hangzhou 310027, China

**Keywords:** cavitation, micro-orifice, microchannel, microfluidic system, computational fluid dynamics (CFD)

## Abstract

Microfluidic systems have witnessed rapid development in recent years. As one of the most common structures, the micro-orifice is always included inside microfluidic systems. Hydrodynamic cavitation in the micro-orifice has been experimentally discovered and is harmful to microfluidic systems. This paper investigates cavitating flow through a micro-orifice. A rectangular micro-orifice with a *l/d* ratio varying from 0.25 to 4 was selected and the pressure difference between the inlet and outlet varied from 50 to 300 kPa. Results show that cavitation intensity increased with an increase in pressure difference. Decreasing exit pressure led to a decrease in cavitation number and cavitation could be prevented by increasing the exit pressure. In addition, the vapor cavity also increased with an increase in pressure difference and *l/d* ratio. Results also show the pressure ratio at cavitation inception was 1.8 when *l/d* was above 0.5 and the cavitation number almost remained constant when *l/d* was larger than 2. Moreover, there was an apparent difference in cavitation number depending on whether *l/d* was larger than 1.

## 1. Introduction

As a significant branch of micro-electro-mechanical systems (MEMS), microfluidic systems have received growing interest in many fields, including fuel cells, medicine, chemical or biomedical analysis, and drug delivery [1,2,3,4,5]. The common microfluidic systems include micropumps, micromixers, microvalves, and lab-on-chip systems [6,7,8,9]. In these microfluidic systems, microchannels—especially micro-orifices—are often encountered and can be used to prevent instabilities and keep a uniform flow distribution or act as network connections, for example, in microchannel evaporators or in corrosion studies [10,11].

It has been established that when static pressure inside machinery reduces to a critical value the nuclei will be triggered and cavitation occurs. Since cavitation may reduce efficiency, generate vibration and acoustic noise, or even cause a catastrophic disaster [12,13], the hydraulic cavitation inside macro machinery has attracted enormous attention from researchers [13,14,15]. Although there are experimental and numerical investigations demonstrating that a microfluidic system with micro-orifices may suffer from the influences of cavitation, there are only a few that concentrate on cavitation inside a microfluidic system until now [16,17,18,19,20,21]. Investigations have mostly focused on explaining the cavitation phenomenon, the effects of turbulent state, and the structural parameters of cavitation characteristics. The principals obtained from macroscale studies may still be applied to a microscale structure [17,18,19,20,21,22,23,24,25,26,27,28,29], however, the turbulent state and scale can affect cavitation characteristics [20].

A high-pressure drop appears when fluid flows through the orifice and cavitation has a high possibility of occurring. There is plenty of research concentrating on cavitation in macroscale orifices [22,23,24,25,26,27]. Orifices with different geometries and a different number of holes have been investigated [22,23,24], along with studies on the ratio of the length and diameter of orifices and boundary conditions [25,26,27]. There is also some research into cavitation flow inside microscale orifices with a low Reynolds number flow [28,29,30,31]. Mishra and Peles [28,29] experimentally investigated cavitating flow in micro-orifices with a rectangular cross-section. The pressure ratio between the inlet and outlet of the microchannel was studied and results showed that cavitating flow pattern was different to macroscale orifices, which were affected by the size of the micro-orifice and microchannel. The released air bubbles behind micro-orifices were also experimentally studied and were found to be related to choking cavitating flow [30,31]. It should be noted that existing studies of cavitation inside micro-orifices were under a laminar flow state, while in microfluidic systems, such as micropumps and microvalves, the flow state is turbulent and the Reynolds number in the orifice can reach up to 25,000 [10,11,32]. Micro-orifices on a scale of tens to hundreds of microns may lead to a different conclusion compared with macroscale orifices. Furthermore, micro-orifices and microchannels often have a rectangular cross-section rather than a circular cross-section when compared with macroscale orifice plates and, thus, the cavitation phenomenon inside micro-orifices needs to be focused upon and thoroughly investigated.

In this paper, the computational fluid dynamics (CFD) method is used to simulate cavitating flow through micro-orifices and the CFD methods are validated through comparison with other experimental results. The simulated velocity and pressure fields, vapor volume fraction, and cavitation number are analyzed and discussed. The baseline micro-orifice has a diameter ratio between the orifice and microchannel of *d/D* = 0.2. The influence of the ratio between the length and the diameter of the micro-orifice; the ratio of inlet pressure and outlet pressure; and the exit pressure have also been investigated. The intent of this work is to provide useful insights for designing MEMS and other microchannels containing micro construction.

## 2. Mathematical Methods

Cavitation occurs when the pressure becomes saturated due to restriction of the micro-orifice, and the single flow becomes a two-phase flow. Due to the high diameter ratio between the microchannel and the orifice [33], there is a remarkable increase in velocity inside an orifice. The average Reynolds number inside an orifice is above 2100, therefore, flow is turbulent inside the orifice. In the inlet and outlet channel, the average Reynolds number is low due to a very low velocity, and flow can be treated as laminar. To capture the vapor emerging at the orifice and save simulation time, the Reynolds-averaged Navier–Stokes (RANS) method with shear stress transport (SST) *k–ω* turbulence model [34] was used, along with a cavitation model based on the Rayleigh–Plesset equation. The geometrical model of the micro-orifice, the turbulence model, and the cavitation model are described below.

### 2.1. Physical Model

**Figure 1:**
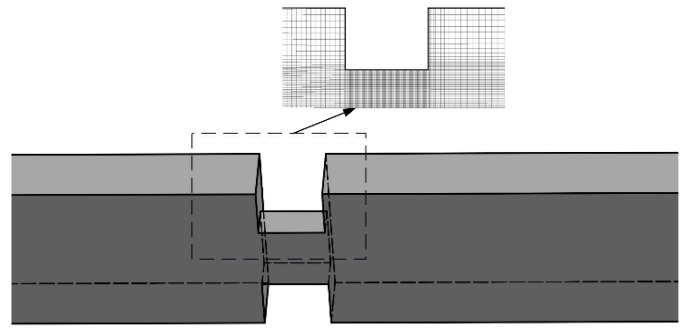
The geometry of the microchannel with micro-orifice.

The scheme of the microchannel with the micro-orifice is depicted in Figure 1. Here, the width and depth of the microchannel are 400 and 300 μm, respectively, and the micro-orifice has an equal width and depth of 160 μm. The length of the microchannel is 5000 μm and the baseline ratio between the length and diameter of the micro-orifice, *l/d*, is 1.

### 2.2. Turbulence Model

Water and water vapor were used as working fluid at a room temperature (22 °C). Continuity and momentum equations needed to be solved, and additional equations were applied to solve the momentum equation. The governing equations and the transport equations of the SST *k*-*ω* turbulence model are defined as follows:

(1) $$\nabla \cdot \left(\rho \vec{u}\right)=0,$$

(2) $$\nabla \left(\rho \vec{u}\vec{u}\right)=-\nabla p+\nabla \left[\mu \left(\nabla \vec{u}+\nabla {\vec{u}}^{T}\right)\right],$$

(3) $$\frac{\partial }{\partial t}\left(\rho k\right)+\frac{\partial }{\partial x_{i}}\left(\rho ku_{i}\right)=\frac{\partial }{\partial x_{j}}\left(\Gamma_{k}\frac{\partial k}{\partial x_{j}}\right)+G_{k}-Y_{k},$$

(4) $$\frac{\partial }{\partial t}\left(\rho \omega \right)+\frac{\partial }{\partial x_{i}}\left(\rho \omega u_{i}\right)=\frac{\partial }{\partial x_{j}}\left(\Gamma_{\omega }\frac{\partial \omega }{\partial x_{j}}\right)+G_{\omega }-Y_{\omega }+D_{\omega }.$$

Here, *ρ* stands for mixture density, $\vec{u}$ stands for mass-averaged velocity, *p* represents pressure, *G_k_* = $-\rho \bar{u_{i}^{\prime }u_{j}^{\prime }\frac{\partial u_{j}}{\partial x_{i}}}$ and *G_ω_* = $\alpha \frac{\omega }{k}G_{k}$ represent the generation of turbulent kinetic energy and specified dissipation rate, respectively, Γ represents the effective diffusivity, *Y_k_* = $\rho \beta^{*}f_{\beta^{*}}k\omega$ represents the dissipation due to turbulence, and *D_ω_* = $\rho \beta f_{\beta }\omega^{2}$ represents the cross-diffusion term. 

### 2.3. Cavitation Model

The commonly used cavitation models are based on the Rayleigh–Plesset equation, and the one applied in this work was proposed by Zwart et al. [35] and has been validated, with sufficient precision, by previous research. In this model, the phase transformation can be expressed as mass changes of vapor as shown in the equation below.

(5) $$\frac{\partial }{\partial t}\left(\alpha \rho_{v}\right)+\nabla \left(\alpha \rho_{v}\vec{V_{v}}\right)=R_{e}-R_{c}$$

Here, *R_e_* represents increases in the vapor mass transfer rate and *R_c_* represents the decrease in the vapor mass transfer rate.

(6) $$R_{e}=F_{vap}\frac{3\alpha_{nuc}\left(1-\alpha_{v}\right)\rho_{v}}{R_{B}}\sqrt{\frac{2\left(P_{v}-P\right)}{3\rho_{l}}},$$

(7) $$R_{c}=F_{cond}\frac{3\alpha_{v}\rho_{v}}{R_{B}}\sqrt{\frac{2\left(P-P_{v}\right)}{3\rho_{l}}}.$$

Here, the subscript *v* stands for the vapor phase, *α* represents the vapor volume fraction, *R_e_* and *R_c_* represent the growth and collapse of bubbles, *P_v_* is the saturated pressure, and the other remaining terms used in this research are empirical constants.

### 2.4. Model Validation

The governing equations, described above, were all solved using the commercial software Fluent. Pressure inlet and pressure outlet boundary conditions were applied. The semi-implicit method for pressure-linked equations (SIMPLE) algorithm was utilized and second order or higher discretization used for the momentum, pressure, turbulent quantities, and vapor transport equations.

**Figure 2:**
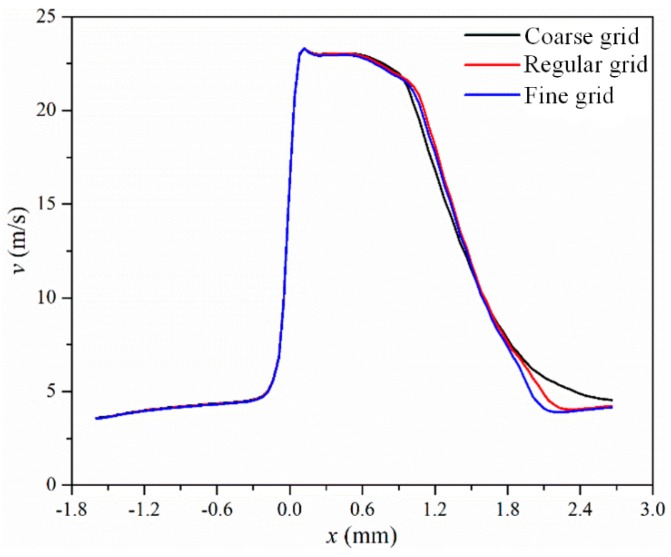
Velocity along the centerline on the symmetry plane under different grids.

**Figure 3:**
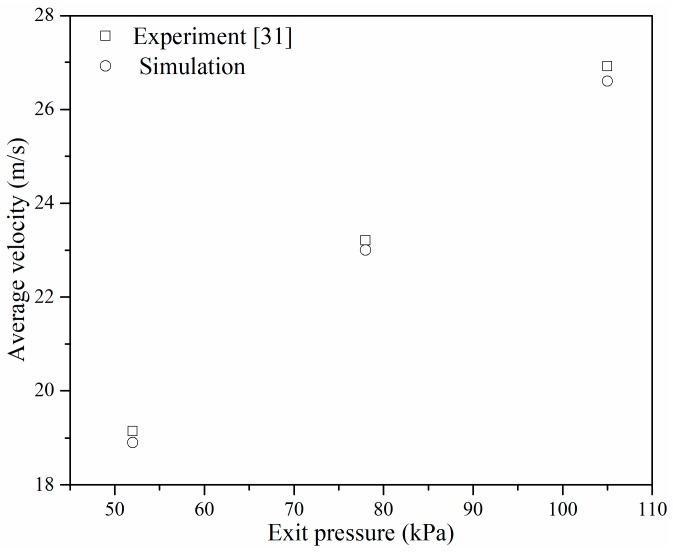
Comparison with experimental results.

A 3D symmetry model was used to save simulation time. A structured grid was created for the flow channel, and a fine grid was used inside the micro-orifice to capture the vapor phase. A boundary layer was also constructed to ensure the value of *y+* was under 1 throughout the simulation. Three kinds of grid were established, and the number of cells was approximately 640,000; 1,260,000; and 2,540,000. Velocity along the centerline on the symmetry plane under different grids is shown in Figure 2 and the maximum vapor volume fraction *α_max_* inside the microchannel under different grids is shown in Table 1. In Figure 2, the maximum velocity lies inside the orifice and the value of maximum velocity is similar to the results of Mishra and Peles [36], as shown in Figure 3. Combining Figure 2 and Table 1, it was found that the effects of the grid can be neglected when the cell count is about 1,260,000; thus, the same grid generation method was applied for all investigated models.

Experiments conducted by Mishra and Peles [36] were selected to validate the methodology used in this study, where the average velocity inside the orifice under different exit pressure was compared, and the results are shown in Figure 3. A good agreement was found when comparing the simulated and experimental results, therefore, the validated methods were utilized in the subsequent study.

## 3. Results and Discussion

The micro-orifice inside a microchannel under different pressure differences, ∆*P*, between the inlet and outlet was numerically investigated, and the effects of exit pressure on cavitation were studied. In addition, the *l*/*d* ratio between the length and diameter of the orifice was changed from 0.25 to 4 to investigate the effects of the length of micro-orifices.

### 3.1. Effects of Pressure Difference

The possibility of cavitation inception increases as the pressure ratio increases. To study the effects of pressure difference on the cavitation phenomenon inside micro-orifices, a micro-orifice with an *l/d* ratio = 1 was chosen. The exit pressure in the microchannel was kept as 100 kPa and the inlet pressure was varied.

The cavitation number is widely used to characterize the cavitation intensity or inception. The cavitation number *σ* inside hydraulic machines and macroscale orifice is defined as follows:

(8) $$\sigma =\frac{P_{2}-P_{v}}{0.5\rho v_{o}^{2}}.$$

Here, *P*_2_ is the upstream and downstream pressure of micro-orifices, and *v_o_* is the mean velocity at the throat of micro-orifices.

**Figure 4:**
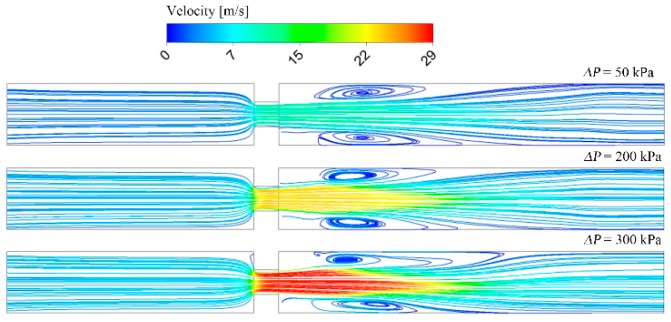
Velocity and streamline on the symmetry plane.

**Figure 5:**
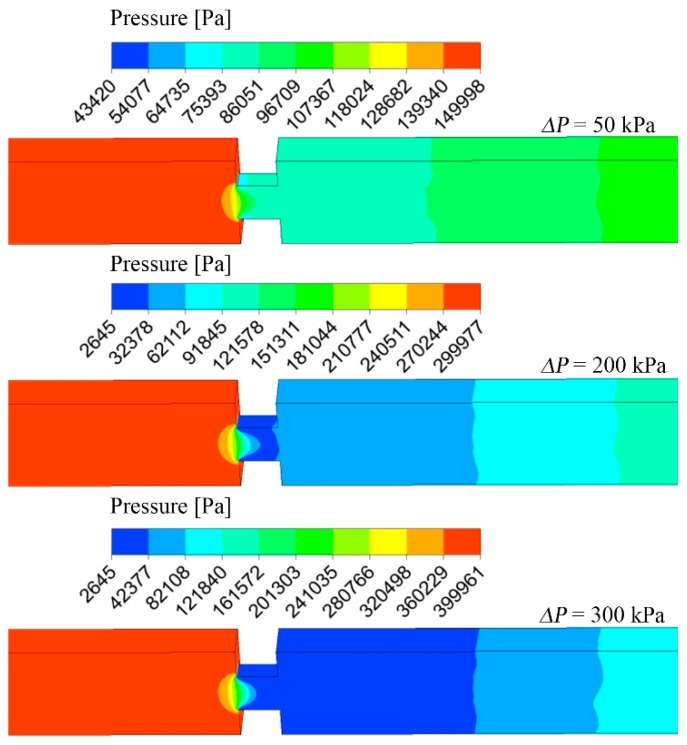
Pressure distribution inside the microchannel.

Velocity streamlines on the symmetry plane of the microchannel are shown in Figure 4, with three different pressure differences. The flow regime under different pressure differences was almost the same and also similar to the flow regime inside macroscale orifices. As pressure difference increased, velocity inside the microchannels increased, especially inside the micro-orifice. If the pressure difference was too high, the pressure inside the micro-orifice can be as low as the saturated pressure and, thus, cavitation occurs. Figure 4 shows that when the pressure difference was 200 kPa the Reynolds number obtained at the inlet and outlet was about 1160, and the Reynolds number inside the orifice was at its maximum with a value of about 4000. Figure 5 shows the pressure distribution inside microchannels, where the pressure on the symmetry and walls are plotted. Under different pressure differences, the pressure distribution was almost the same, which coincides with the velocity distribution. When the pressure difference is 50 kPa, the minimum pressure inside the microchannel is larger than the saturated vapor pressure and, thus, there is no cavitation. While the pressure difference was above 200 kPa, an obvious low-pressure zone that was equal to saturated pressure was found on the outer wall at the entrance of the micro-orifices. When the pressure difference rose to 300 kPa, a larger low-pressure zone was observed. The pressure difference at cavitation inception was 80 kPa, therefore, when the exit pressure is 100 kPa the inlet pressure should be smaller than 180 kPa to avoid the occurrence of cavitation.

**Figure 6:**
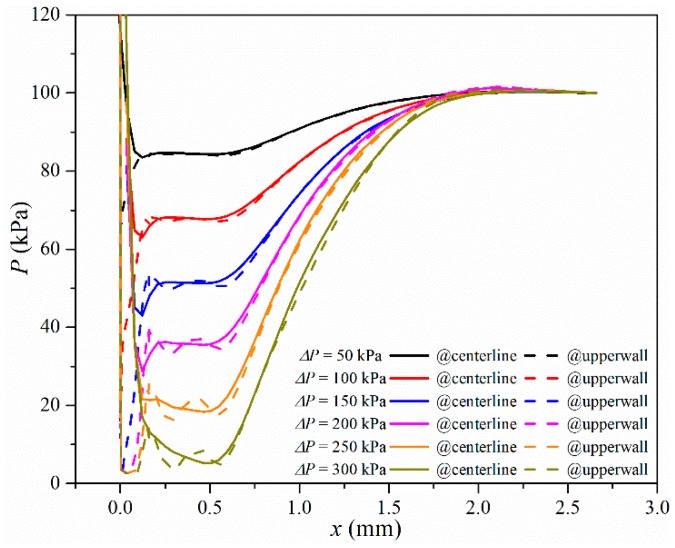
Pressure along two different locations on the symmetry plane.

To specify the pressure value under different pressure differences, pressure profiles on the centerline and outer upper wall on the symmetry plane are shown in Figure 6. As the pressure difference increased the pressure value decreased after the entrance of the micro-orifice and there was pressure recovery downstream of the micro-orifices. When the pressure difference was 50 kPa, all pressure was higher than saturated pressure. When the pressure difference was larger than 150 kPa, a small low-pressure region began to appear, and low pressure equal to the saturated pressure appeared on the upper wall of the micro-orifices after a pressure difference of 200 kPa. When the pressure difference was larger than 300 kPa, there was saturated pressure on the entire upper wall. As the pressure difference continued to increase, the pressure value downstream of the micro-orifice may also decrease to the saturated pressure, which can be found in Figure 6, with a pressure difference equal to 350 kPa.

**Figure 7:**
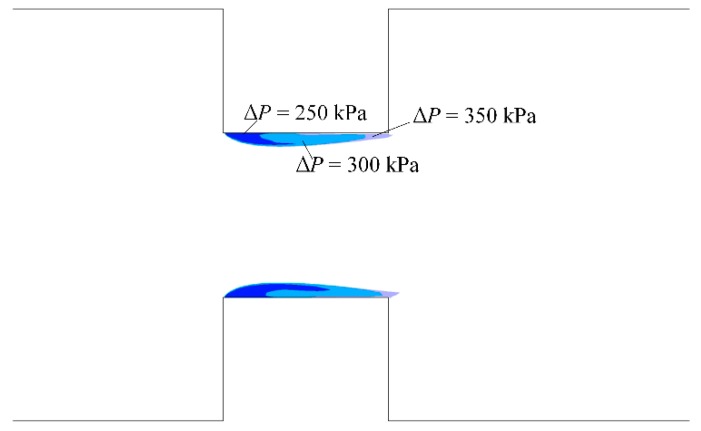
Vapor volume fraction on the symmetry plane vs. different pressure differences with *l*/*d* = 1.0.

Figure 7 shows the vapor distribution on the symmetry plane when the vapor volume fraction was above 0.5, which clearly shows the development of vapor cavity as pressure difference increased. The vapor cavity inside micro-orifices had a similar outer contour, and a large difference in the shape of the vapor cavity appeared between ∆*P* = 250 kPa and ∆*P* = 300 kPa. When the pressure difference was 350 kPa, the entire micro-orifice wall was covered by the vapor cavity. A comparison of the vapor cavity under different pressure differences indicates that pressure difference has a significant influence on cavitation inception and intensity inside the microchannel with micro-orifices.

### 3.2. Effects of Exit Pressure

Exit pressure was varied to study its effect on cavitation inside the micro-orifice. The same pressure difference and pressure ratio between the inlet and outlet were chosen, while the exit pressure was varied at 40, 50, and 100 kPa.

**Figure 8:**
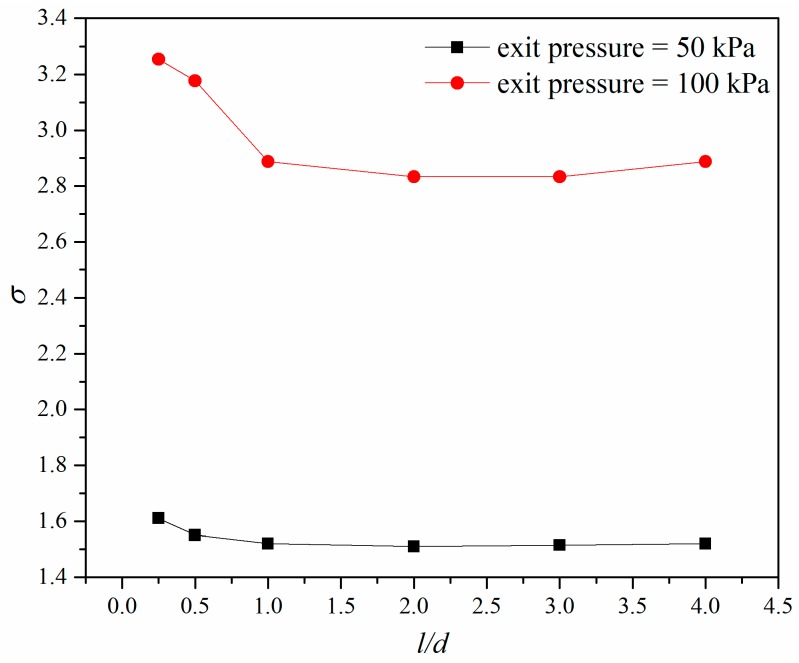
Cavitation number under different exit pressure for the same pressure difference.

When the pressure difference was the same, the cavitation number under different exit pressures and different *l/d* ratios between the length and diameter of the micro-orifices is shown in Figure 8. Here, the pressure difference was kept at 50 kPa. From Figure 8, it can be seen that cavitation occurs when there is a low exit pressure, as no cavitation was found inside micro-orifices when the exit pressure was high. The pressure ratio was 1.5 for high exit pressure and 2 for low exit pressure, therefore, it can be inferred that there is a relationship between cavitation inception and the pressure ratio between the inlet and outlet.

**Figure 9:**
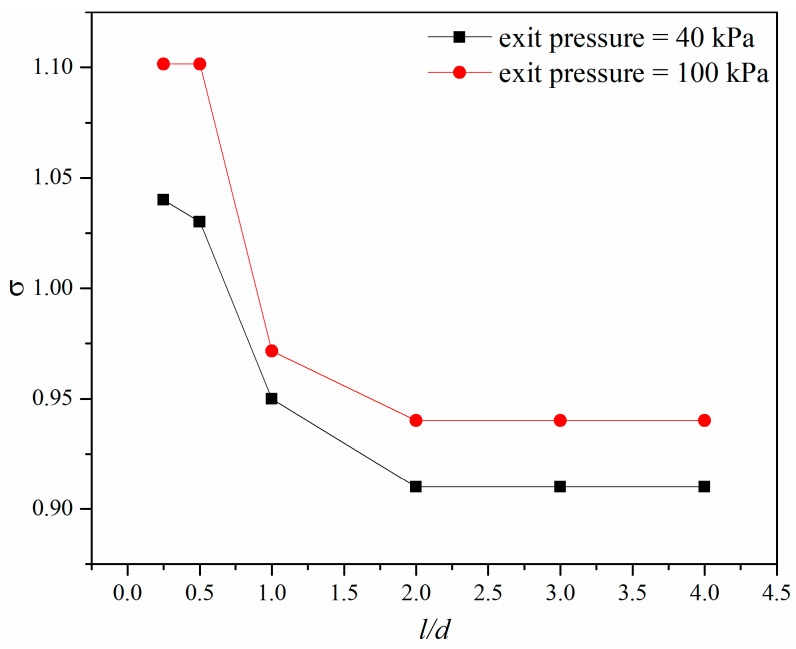
Cavitation number under different exit pressures for the same pressure ratio.

Figure 9 shows the cavitation number under different exit pressure when the pressure ratio was 2.5. It was found that when *l/d* was above 2.0 the cavitation number remained unchanged, and when *l/d* was smaller than 2.0 the cavitation number decreased with the increase of *l/d*. Under the same pressure ratio, the micro-orifice with lower exit pressure had a lower cavitation number, which means that decreasing the exit pressure will increase the cavitation intensity of micro-orifices.

### 3.3. Effects of Micro-Orifice Length

The length of the micro-orifice has effects on its throttling ability and, thus, the effects of the length of micro-orifices on the cavitation phenomenon need to be investigated. Microchannels with different *l/d* ratios between the length and diameter of the micro-orifice were studied and the pressure difference was also varied.

**Figure 10:**
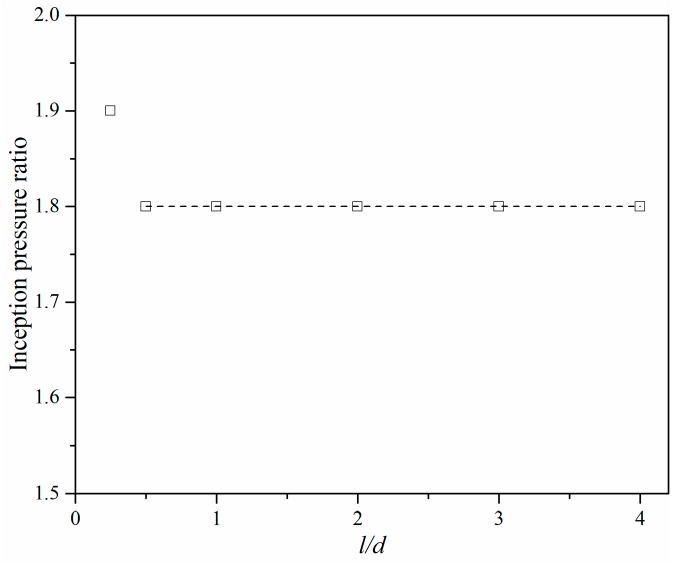
Pressure ratio vs. ratio between length and diameter at cavitation inception.

The minimum pressure inside the microchannel was treated as the criterion to judge whether cavitation inception occurred. If the minimum pressure was saturated, the relative pressure ratio was regarded as the incipient pressure ratio. Figure 10 shows the pressure ratio under different *l/d* ratios when cavitation inside micro-orifices has just appeared. Figure 10 shows that the inception pressure ratio of the micro-orifice is basically the same if the *l/d* ratio is above 0.5, however, when *l/d* is smaller in value, for example, 0.25, the inception pressure ratio is slightly higher and the increment is about 5%. The relationship between the inception pressure ratio and *l/d* of the micro-orifice was different to the investigated macroscale orifice [25], where the inception pressure ratio had a large variation if *l/d* was smaller than 2.0. In addition, the inception pressure ratio of a micro-orifice was also different to the macroscale orifice with the same exit pressure but the inception pressure ratio was slightly smaller, however, the cavitation number was larger than in a macroscale orifice [27].

**Figure 11:**
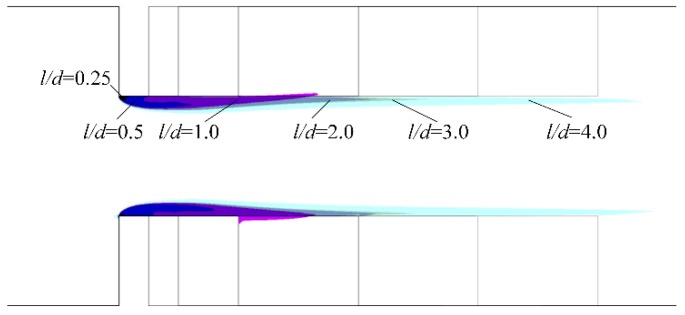
Vapor volume fraction on the symmetry plane vs. different *l/d* when the pressure difference is 300 kPa.

**Figure 12:**
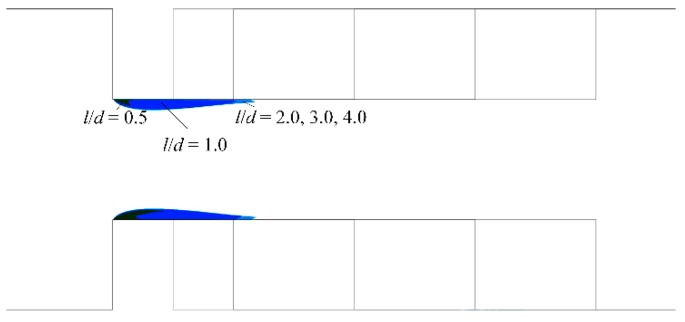
Vapor volume fraction on the symmetry plane vs. different *l*/*d* with α > 0.5.

Though *l/d* does not affect cavitation inception in a micro-orifice, a larger *l/d* can delay pressure recovery and enlarge the low-pressure zone. Figure 11 shows the vapor cavity on the symmetry plane under different *l/d* when the pressure difference is 300 kPa, and the vapor cavity area can be seen to increase with the increase of *l/d*. There are studies that investigate cavitation characteristics when the vapor volume fraction is above 0.5 because, under these conditions, the generated cavitation may lead to serious damage to hydraulic machines. The vapor distribution on the symmetry plane when the vapor volume fraction is above 0.5 is shown in Figure 12. As shown in Figure 12, when *l/d* equals 0.25, the vapor cavity on the symmetry plane is barely visible. With an increase in *l/d*, a vapor cavity became apparent on the symmetry plane. Although the vapor cavity inside the micro-orifice had a similar outer contour, the vapor cavity region increased with the increasing *l/d* if *l/d* was smaller than 2.0. When *l/d* was between 2.0 and 4.0, the vapor cavity region on the symmetry plane was almost the same, and the difference indistinguishable. This was also different from the vapor distribution on the symmetry plane in a macroscale orifice, where a large difference of vapor cavity existed between different length *l/d* ratios in macroscale orifices [27].

The discharge of orifice is related to the pressure difference between the inlet and the outlet, and can be defined as follows:

(9) $$Q=CA_{o}\sqrt{2\Delta P\rho^{-1}{\left(1-{\left(A_{o}/A_{c}\right)}^{2}\right)}^{-1}}.$$

Here, *Q* represents the volumetric flow rate, *A_c_* represents the cross-sectional area of the microchannel, *A_o_* represents the cross-sectional area of the micro-orifice, and *C* is the discharge coefficient.

**Figure 13:**
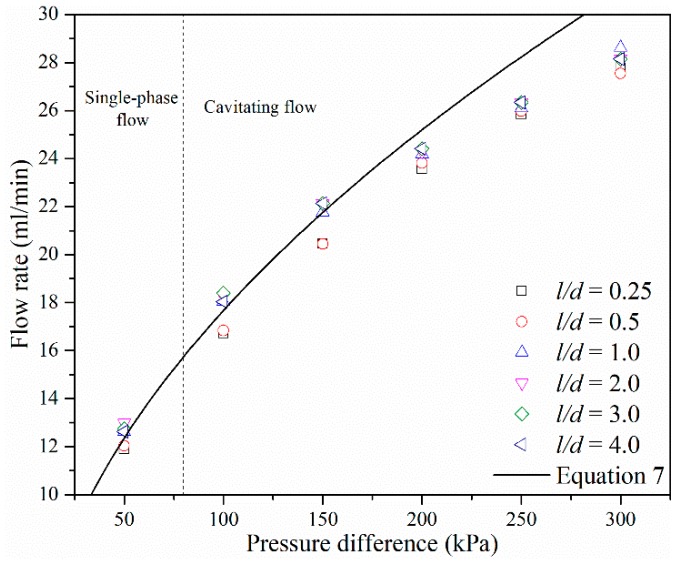
Flow rate vs. pressure drop for different *l/d* ratios.

The relationship between the volumetric flow rate and pressure difference between the inlet and outlet should be a quadratic relationship as described in Equation (9). The simulated flow rate *Q* and ∆*P* under different *l/d* are plotted in Figure 13. As shown in Figure 13, after cavitation has occurred, the relationship between the flow rate and pressure difference starts to deviate from Equation (9). As the pressure difference increased, the increment of flow rate decreased and flow rate trends become constant, which is consistent with the experimental results of Mishra and Peles [36]. The effects of *l/d* on the flow rate of micro-orifices depend on the range of *l/d* values. If *l/d* is larger or equal to 1, flow rate under different *l/d* is almost constant. If *l/d* is smaller than 1, the flow rate under different *l/d* is also constant but the value is smaller compared to the one under larger *l/d*. 

**Figure 14:**
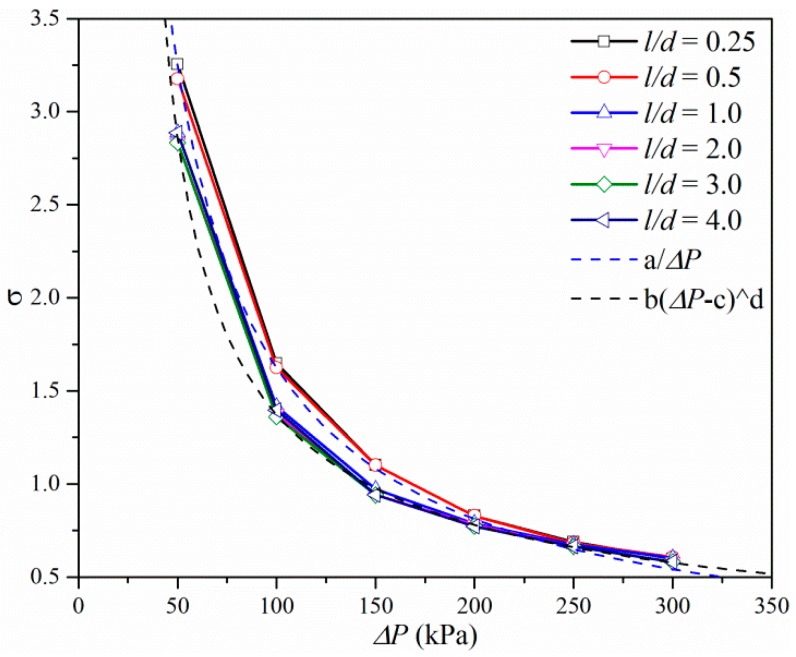
Cavitation number vs. pressure ratio and *l/d*.

Figure 14 shows the cavitation number under different pressure differences and different *l/d*. The cavitation number can be seen to decrease as the pressure difference increases, which means that cavitation intensity is high under a higher pressure ratio. The cavitation number has a reciprocal relationship with the square of the volumetric flow rate according to Equation (8). The pressure difference between the inlet and = outlet of the microchannel has a linear relationship with the square of the volumetric flow rate according to Equation (9). Owing to the linear relationship between the flow rate and velocity, the cavitation number may have a reciprocal relationship with the pressure difference. As shown in Figure 13, when *l/d* is smaller than 1 the cavitation number has a reciprocal relationship with ∆*P* as the outlet pressure is constant. When *l/d* is larger than 1 the cavitation number only has an exponential relationship with ∆*P*. The results demonstrate that longer lengths micro-orifice can increase the cavitation intensity and flow rate of micro-orifices.

## 4. Conclusions

The computational fluid dynamics method was utilized to investigate cavitating flow inside micro-orifices. The effects of the pressure difference between the inlet and outlet of the microchannel, the exit pressure of the microchannel, and the ratio between the length and diameter of a micro-orifice on cavitation were investigated. The numerical models were validated by comparing the experimental results, which demonstrated that higher pressure differences led to higher cavitation intensity. With an increase in pressure difference, the vapor cavity region increases, and the entire micro-orifice wall can be covered by vapor. In addition, cavitation intensity also increased with decreasing exit pressure. The inception pressure ratio remained constant at 1.8 when *l/d* was larger than 0.5, and the inception pressure ratio was a little higher when *l/d* was smaller than 0.5. As for the increase of *l/d*, the vapor cavity region increased under the same pressure difference. Moreover, when the pressure difference was small the cavitation number under large *l/d* was smaller than the cavitation number under small *l/d*. The cavitation number was almost the same when *l/d* was above 1. While the pressure difference was high, the cavitation number under all *l/d* was almost the same. Therefore, it is recommended that the maximum *l/d* of a micro-orifice is 1, above which cavitation intensity is high but the flow rate barely changes, and a higher exit pressure should be applied.

## Figures and Tables

**Table 1:** Maximum vapor volume fraction under the different grid.

Grid	Coarse	Regular	Fine
*α_max_*	0.937	0.963	0.965
